# UNRAVELING THE THREADS OF DEMENTIA PERCEPTION AMONG OLDER ASIAN INDIAN IMMIGRANTS IN THE UNITED STATES

**DOI:** 10.1093/geroni/igae098.3459

**Published:** 2024-12-31

**Authors:** Malinee Neelamegam, Shilpa Patil, RoiSan Nhpang, Stacey Griner

**Affiliations:** University of North Texas Health Science Center, Fort Worth, Texas, United States; University of North Texas Health Science Center, Fort Worth, Texas, United States; University of North Texas Health Science Center, Fort Worth, Texas, United States; University of North Texas Health Science Center, Fort Worth, Texas, United States

## Abstract

The Asian Indian (AI) population, the second-largest immigrant group in the US, includes a substantial proportion aged 50 and older. Despite facing cognitive risks, they’re underrepresented in aging research. Our study delves into dementia perceptions among older AI immigrants in the US. We conducted in-depth interviews with 12 Asian Indian immigrants aged 50 and above in the Dallas-Fort Worth Metroplex. Guided by a semi-structured interview guide, we explored their perceptions of dementia and aging in the US. Thematic analysis, utilizing both predetermined and emerging codes, was employed to analyze the data. Of the participants, the majority were women (n=21), with an average age of 68.9 years (SD=5.3) and an average length of stay in the US of 35.1 years (SD=13.1). Alarmingly, there was a notable lack of awareness regarding cognitive aging and dementia. Emerging themes included concerns about the financial burden of screening and treatment, societal acceptance, and access to support services. Moreover, participants’ immigration journeys significantly influenced their social networks and caregiving choices as they aged. Addressing the expense of care, enhancing caregiving resources, and addressing the low awareness of brain health among older AI immigrants in the US are crucial focal points for future research. These findings can inform culturally tailored initiatives and interventions aimed at dementia and aging within South Asian communities.

